# Effect of calcium hydroxide removal protocols on push-out bond strength of hydraulic calcium silicate sealers: an *in vitro* study

**DOI:** 10.7717/peerj.21236

**Published:** 2026-05-14

**Authors:** Özlem Sivas Yılmaz, Cangül Keskin, Ayşegül Güven Kömürcü

**Affiliations:** 1 Department of Endodontics, Faculty of Dentistry, Abant Izzet Baysal University, Bolu, Turkey; 2 Department of Endodontics, Faculty of Dentistry, Ondokuz Mayis University Samsun, Turkey

**Keywords:** Calcium hydroxide, Hydraulic calcium silicate sealer, Intracanal dressing

## Abstract

**Background and Objectives:**

This study aims to evaluate the push-out bond strength of a hydraulic calcium silicate sealer (HCSS) (AH Plus Bioceramic; Dentsply Sirona, Tulsa, OK, USA), to dentin following calcium hydroxide (Ca(OH)_2_) removal using different irrigation protocols and to assess the sealer-dentin adhesive interface.

**Materials and Methods:**

Forty-four single-rooted human teeth were prepared, standardized to 16 mm in length, and instrumented with ProTaper Next to an X5 size. After final irrigation, the canals were filled with Ca(OH)_2_ for two weeks. The specimens were divided into four groups according to the irrigation protocol: distilled water (control), ethylenediaminetetraacetic acid (EDTA) combined with NaOCl, citric acid, and glycolic acid. The canals were subsequently dried and filled with HCSS, a premixed hydraulic calcium silicate sealer, using an X5 gutta-percha cone. The specimens were stored at 37 °C and 100% humidity for 7-day before testing. Push-out bond strength was measured using a universal testing machine, and failure modes were evaluated under a stereomicroscope.

**Results:**

Glycolic acid exhibited the highest overall bond strength, particularly in the coronal third (*p* < .05), compared to distilled water. EDTA + NaOCl and citric acid demonstrated similar bond strength values, both statistically superior to distilled water (*p* < .05), but not significantly different from glycolic acid (*p* > .05). Failure mode analysis revealed predominantly cohesive failures, with no statistically significant variation in failure modes among irrigation protocols or root canal thirds (*p* > .05).

**Conclusion:**

The irrigation protocol used for Ca(OH)_2_ removal significantly influenced the push-out bond strength of HCSS. Glycolic acid demonstrated the highest bond strength, particularly in the coronal third, while EDTA + NaOCl and citric acid also enhanced bonding compared to the control group.

## Introduction

The success of root canal treatment (RCT) largely depends on the quality of the seal established along the root canal system, which is achieved by using a root canal sealer that adheres effectively to dentinal walls (Gillen et al., 2011). Epoxy resin-based sealers, such as “the gold standard” AH Plus, are widely utilized in clinical practice due to their established mechanical and adhesive properties, contributing to treatment success (Donnermeyer et al., 2019). Recently, developments in material science have introduced hydraulic calcium silicate sealers (HCSSs) aimed at enhancing biocompatibility, sealing ability, and handling properties (Aminoshariae, Primus & Kulild, 2022). HCSSs incorporate components such as tricalcium silicate, zirconium dioxide, dimethyl sulfoxide, and lithium carbonate. The calcium silicate content reportedly releases calcium ions, supporting bioactivity and potential apatite formation along the dentinal interface, which may enhance mineralization and tissue compatibility (Sanz et al., 2022; De Souza et al., 2023). This formulation is claimed to be moisture-compatible, setting in humid conditions similar to the root canal environment (AH Plus Brochure, 2026). Furthermore, it is suggested to expand slightly upon setting, which may contribute to sealing effectiveness by reducing voids and microleakage.

In laboratory studies, HCSSs have demonstrated comparable or slightly lower push-out bond strength (PBS) than epoxy resin–based sealers such as AH Plus. (Al-Hiyasat & Alfirjani, 2019). However, the bond strength of HCSSs showed significant increase due to its bioactive properties when exposed to body fluids, promoting hydroxyapatite formation along the dentin interface (Abu Zeid & Alnoury, 2024).

An essential parameter for evaluating the clinical performance of a sealer is its bond strength to root dentin, which is associated with the longevity of the endodontic seal (International Organization for Standardization, 2012). In RCT, calcium hydroxide (Ca(OH)_2_) is frequently employed as an intracanal dressing due to its antimicrobial effects and ability to neutralize bacterial endotoxins (Bedran et al., 2020). However, residual Ca(OH)_2_ within the canal can potentially impair the bonding capacity of subsequently applied sealers (Vilela et al., 2011). Effective irrigation protocols are thus recommended to eliminate Ca(OH)_2_ residues using chelators such as ethylenediaminetetraacetic acid (EDTA), citric acid or glycolic acid (Keskin, Keleş & Sarıyılmaz, 2021; Da Silva et al., 2011).

Studies (Savaris et al., 2024; Prado, Simão & Gomes, 2013) evaluating the resin-based sealers have demonstrated that specific irrigation protocols significantly influence (PBS) to dentin. For instance, the sequential use of EDTA followed by NaOCl has been shown to enhance bond strength and promote a uniform adhesive interface (Savaris et al., 2024). In addition to conventional chelating agents such as EDTA and citric acid, glycolic acid has recently been proposed as an alternative irrigation solution in endodontics (Dal Bello et al., 2019; Souza et al., 2024; Yanık & Turker, 2024; Calt & Serper, 2002; De Andrade Marafiga et al., 2022). This is primarily due to several limitations of EDTA such as its potential to cause excessive dentinal erosion, and its low biodegradability in the environment. Chemically, prolonged exposure to EDTA can induce excessive dentinal erosion and reduce microhardness, which may compromise the structural integrity required for hydraulic calcium silicate sealers (Baldasso et al., 2017). EDTA is also known for its high environmental persistence and poor biodegradability. Its potential to mobilize heavy metals in water systems led the exploration of more biocompatible and biodegradable alternatives, such as organic alpha-hydroxy acids (Dal Bello et al., 2019; Xie, Healy & Russell, 2007).

Glycolic acid is an α-hydroxy acid with chelating properties and has been shown to effectively remove smear layer and Ca(OH)_2_ residues, while exhibiting a lower erosive effect on dentin compared with EDTA (Dal Bello et al., 2019; De Andrade Marafiga et al., 2022). Previous studies have demonstrated its efficacy in removing calcium hydroxide from complex root canal irregularities and simulated resorption cavities (Keskin, Keleş & Sarıyılmaz, 2021). However, despite emerging evidence regarding its cleaning efficacy, data concerning the interaction between glycolic acid–treated dentin surfaces and HCSSs remain limited.

Although several studies have investigated the influence of Ca(OH)_2_ removal protocols on the bond strength of resin-based sealers (Savaris et al., 2024; Prado, Simão & Gomes, 2013), evidence regarding their effects on HCSSs has not been remains limited.. Crucially, the bonding mechanism of HCSSs differs fundamentally from that of epoxy resin–based sealers. While resin sealers rely primarily on macromechanical interlocking and a dry substrate for polymerization, HCSSs are hydrophilic materials that require moisture for their hydration reaction and form a chemical bond *via* the precipitation of hydroxyapatite precursors (Aminoshariae, Primus & Kulild, 2022; Al-Hiyasat & Alfirjani, 2019; Abu Zeid & Alnoury, 2024). Consequently, standard irrigation protocols optimized for resin sealers, which focus heavily on aggressive demineralization to expose collagen fibrils may not be ideal for HCSSs. Excessive demineralization could potentially compromise the mineral content required for the bioactive interaction of HCSSs. Therefore, findings obtained from resin-based sealers cannot be directly extrapolated to HCSSs, highlighting the need for studies specifically addressing Ca(OH)_2_ removal protocols in relation to hydraulic calcium silicate sealer–dentin bonding. This study aims to assess the PBS of HCSS following calcium hydroxide Ca(OH)_2_ removal with various irrigation protocols and to examine the quality of the sealer-dentin interface. The null hypothesis states that different irrigation protocols for calcium hydroxide Ca(OH)_2_ dressing removal (distilled water, EDTA + NaOCl, citric acid, or glycolic acid) do not significantly affect the PBS of HCSS to root canal dentin.

## Materials and Methods

The study protocol was approved by the Bolu Abant Izzet Baysal University’s non-interventional clinical research ethics board (approval number: 2024/116). Written informed consent was obtained from all volunteers prior to the collection of the extracted teeth for use in this study. *A priori* sample size was calculated based on PBS values of a previous study having an effect size (0.61) with an alpha type error of 0.05 and a power beta of 0.95 (G*Power 3.1 for Mac; Heinrich Heine, Universitat Dusseldorf, Düsseldorf, Germany) indicated 11 samples per group would require to achieve similar effect size (Savaris et al., 2024).

### Sample preparation and experimental groups

A total of 44 single-rooted premolars were selected from a pool of extracted premolars collected for this study. Teeth were cleaned using periodontal curettes (15/16; Hu-Friedy Co, Chicago, IL, USA) and stored in 0.1% thymol solution (pH = 7) at 4 °C. Radiographs were taken in mesiodistal and buccolingual planes to confirm presence of a single round root and single canal. Round shaped roots (≤1.5 ratio between the buccolingual and mesiodistal root distance) with a mature apex were selected. After screening under a stereomicroscope at 4x magnification, six teeth with defects such as calcification, cracks, vertical fractures, resorption were excluded.

The crowns of the teeth were trimmed from the cementoenamel junction using a diamond bur (Kare Dental, Türkiye). The root lengths of all teeth were adjusted to 16 mm. The working length (WL) was determined to be one mm shorter than the apical foramen. ProTaper Next rotary file system (Dentsply Sirona, Ballaigues, Switzerland) was used for the preparation of the specimens. All files were used in order (X1, X2, X3, X4, and X5) and the final preparation size was X5 (50.05). The instruments were operated with continuous rotary brushing motion at a speed of 300 rpm and a torque of 2 Ncm using (VDW Gold, VDW, Munich, Germany). Between each file change, the root canals were irrigated with six mL of 5.25% NaOCl using a 31 G irrigation needle (NaviTip; Ultradent, South Jordan, UT). Irrigation was performed using back-and-forth movements for 1 min, two mm short of the WL. Final irrigation was carried out with six mL of 17% EDTA and an additional six mL of 5.25% NaOCl.

Root canals were dried using X5 paper points (Dentsply Sirona, Charlotte, NC, USA). Ca(OH)_2_ (Ultracal XS, Ultradent Products, South Jordan, UT, USA) was placed in the canal, two mm shorter than the WL. The coronal surfaces of the roots were sealed using a temporary material (Coltosol F, Coltene/Whaledent, Allstetten, Switzerland), and the specimens were kept at 37 °C with 100% humidity for a duration of 2-weeks.

After 2-weeks, the specimens were randomly divided into 4 groups (*n* = 11):

**Group 1 –(Control Group)** –six mL distilled water

**Group 2 –(EDTA + NaOCl)** –three mL 17% EDTA and three mL 5.25% NaOCl

**Group 3 –(Citric acid)** –six mL 10% citric acid

**Group 4 –(Glycolic acid)** –six mL 5% glycolic acid

Irrigation was adjusted to be two mm short of the WL and activated with back-and-forth movements using a 31 G irrigation needle. During the first three mL of irrigation, irrigation was activated for 30 s using a 15 K type file (Dentsply Sirona, Ballaigues, Switzerland). Subsequently, the final shaping file (X5) was reintroduced to the working length in a brushing motion. This step was performed not for further enlargement, but to mechanically disrupt the dense calcium hydroxide mass and ensuring the apical fit of the master cone. The remaining three mL of irrigation was performed using only the relevant irrigation solution, without any additional procedures. Root canals were dried using X5 paper points.

Root canal obturation was performed using AH Plus Bioceramic Sealer (Dentsply Sirona, Tulsa, OK, USA) and gutta-percha using a standardized lateral compaction technique. The premixed, injectable hydraulic calcium silicate sealer was delivered directly into the root canal using the manufacturer-supplied 24-gauge capillary tip. The tip was inserted into the coronal two-thirds of the canal without binding, and the sealer was injected in a slow, continuous retrograde manner until it was visible at the canal orifice, ensuring no air entrapment.

An X5 master gutta-percha cone, coated with a thin layer of sealer, was slowly inserted to the full working length (WL) to minimize hydraulic pressure. Lateral compaction was then performed using a size 30 stainless steel finger spreader (Dentsply Sirona), which was inserted two mm shorter than the WL adjacent to the master cone. Accessory gutta-percha cones (size 25/.02) were coated with sealer and inserted into the space created by the spreader. This process was repeated until the spreader could not penetrate beyond the coronal third of the canal. Excess gutta-percha was removed at the cemento-enamel junction using a heated plugger, and the coronal seal was established with a temporary restorative material. All obturation procedures were performed by the same operator following an identical standardized protocol for all specimens. The specimens were kept at 37 °C and 100% humidity for 7-days.

### PBS test

Sections one mm thick were obtained from each root using a double-sided diamond disk (South Bay Technology, San Clement, CA). The sections were obtained with a metallographic cutter (Isomet1000, Buehler, Lake Forest, IL, USA) at 350 rpm and thickness of each section was measured with a digital caliper with an accuracy of 0.01 mm. A total of six sections were obtained, with two sections taken from each third: cervical, middle, and apical. One of every two slices obtained from the cervical, middle, and apical thirds was used for fracture testing in the Universal Test Machine (Instron Model 4444; Instron Corp, Canton, MA, USA).

A cylindrical piston with an active tip was selected according to the root canal diameter verified by measurement by digital caliper. The force was applied to the sections at a speed of 0.5 mm/min in the corona-apical direction until filling dislocation occurred with the tip selected according to measured canal diameter (0.5 mm for apical, 0.7 mm for middle and 1.0 mm for coronal slices, respectively). The sealer bonding area was calculated and used to divide the load at failure in N, to determine bond strength (El-Ma’aita, Qualtrough & Watts, 2013). The remaining dentin slices from the cervical, middle, and apical thirds were prepared for adhesive interface analysis. The specimens were fixation and dehydration for scanning electron microscope (SEM) preparation. After this process, the specimens were embedded in epoxy resin and polished with abrasive silicon sandpapers with grits from 400 to 3,000 and were immersed in HCl for 30 s for demineralization and then treated with a 2% NaOCl solution for 10 min. The specimens were then washed with distilled water and immersed in ultrasonic bath. After drying, the samples were coated with gold/palladium. The analysis was conducted using SEM device (JEOL JSM 6390 LV, Akishima, Japan) operating at 10 kV, with magnifications of X20, X100, X250, X500, and X4000. Photomicrographs were obtained for visualization of interfaces qualitatively for the homogeneity and continuity of the filling. SEM analysis was performed for qualitative evaluation of the sealer–dentin interfacial morphology.

### Failure modes analysis

Push-out tested slices were examined under a stereomicroscope (SMZ18; Nikon Instruments Inc., Melville, NY, USA) at 40x magnification. Failure modes were categorized as:

Adhesive Failure: Sealer separated from the dentin surface

Cohesive Failure: Fractured sealer with partial detachment from dentin surface

Mixed Failure: Combination of adhesive and cohesive failure showing partial detachment with some areas still covered by sealer

### Statistical analysis

Kruskal-Wallis H test with Mann Whitney U tests were used to compare the POBS values among Ca(OH)_2_ removal irrigation protocols for each root canal third after Shapiro–Wilk test indicated the data showed non-uniformity with a normal distribution. The chi-square test was conducted to determine if the distribution of fracture types (adhesive, cohesive, mixed) is significantly associated with the irrigation protocol used. All statistical analyses were performed with SPSS V21 (IBM Corp., Armonk, NY, USA) with 5% significance threshold.

## Results

The PBS values for each irrigation protocol across the coronal, middle, and apical thirds are summarized in Table 1. Statistical analysis revealed significant differences both among irrigation protocols and root canal thirds. Glycolic acid showed significantly higher total bond strength compared to distilled water, particularly in the coronal and apical thirds (*p* < .05). EDTA + NaOCl and citric acid demonstrated similar total bond strengths (*p* > .05), both statistically superior to distilled water (*p* < .05) but not significantly different from glycolic acid (*p* > .05).

**Table 1:** Push-out bond strength values (mean ± SD) according to irrigation protocols and root thirds (MPa).

**Groups**	**Root canal thirds**			**Total**
	**Coronal**	**Middle**	**Apical**	
**Distilled water**	3.01 ± 0.62^Aa^	3.80 ± 0.36^Aa^	1.95 ± 0.42^Aa^	2.91 ± 0.48^a^
**17% EDTA + 5.25% NaOCl**	3.60 ± 1.23^Aa^	7.0 ± 1.37^Bb^	3.1 ± 1.02^Ba^	4.08 ± 1.06^ab^
**10% Citric acid**	4.33 ± 0.40^Aa^	7.75 ± 1.16^ABb^	4.08 ± 1.10^Ba^	5.38 ± 0.93^ab^
**5% Glycolic acid**	9.97 ± 1.51^ABb^	7.01 ± 2.85^Ab^	4.43 ± 0.14^Ba^	7.66 ± 1.33^b^

**Notes.**

Different superscript capital letters in the same line indicate statistically significant difference among root thirds (*p* < .05). Different superscript letters in the same column indicate statistically significant difference among irrigation groups (*p* < .05).

**Figure 1:**
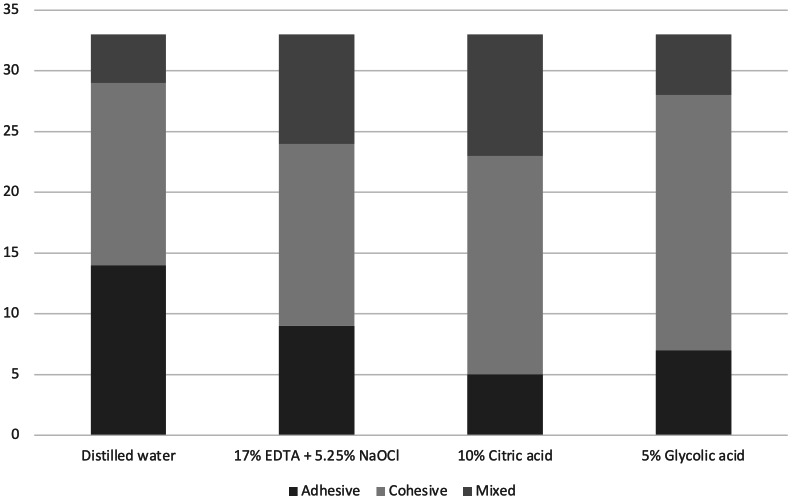
Distribution of failure modes within each experimental group.

The failure modes observed were predominantly cohesive within the sealer, with some mixed failures (Fig. 1). No significant variation in failure mode was noted between groups or root canal thirds (*p* > .05) (Table 2).

**Table 2:** Distribution of fracture types (adhesive, cohesive, and mixed) under different irrigation protocols.

**Groups**	**Fracture type**		
	**Adhesive**	**Cohesive**	**Mixed**
**Distilled water**	14	15	4
**17% EDTA + 5.25% NaOCl**	9	15	9
**10% Citric acid**	5	18	10
**5% Glycolic acid**	7	21	5

**Figure 2:**
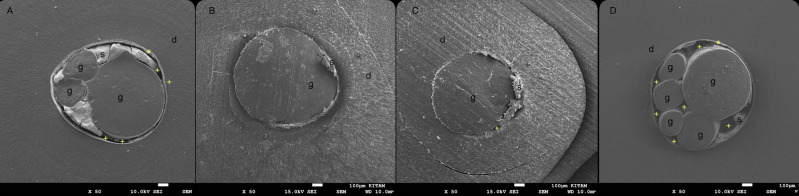
Photomicrographs of thin slice cross sections from the middle thirds (X50). Representative SEM photomicrographs of thin slice cross sections from the middle thirds (X50). (A) Control group, (B) glycolic acid group, (C) EDTA + sodium hypochlorite group and (D), citric acid group. g: gutta percha, d: dentin, s: sealer, * * indicates areas suggestive of residual material along the canal wall, + indicates voids observed at the dentin–sealer interface.

**Figure 3:**
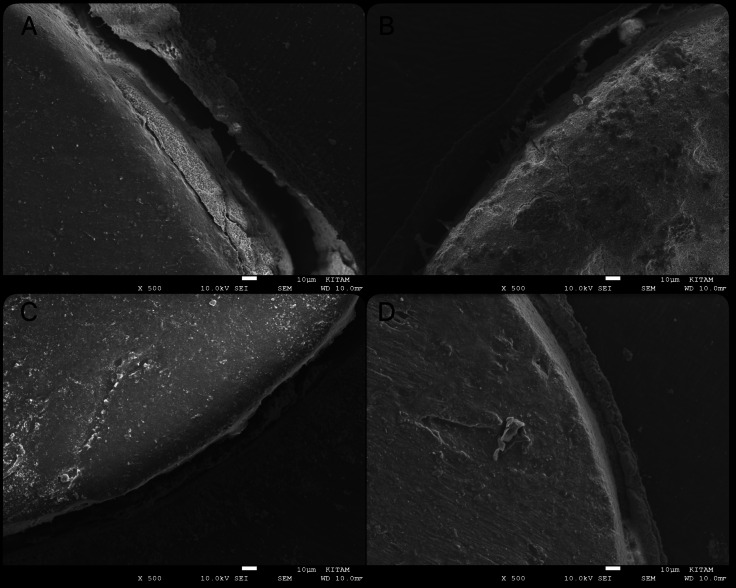
Photomicrographs of the bond interfaces of filling material to root dentin in middle sections (X500). Representative SEM images showing the sealer–dentin interfacial morphology in middle sections (X500) (A) control group, (B) glycolic acid group, (C) EDTA + sodium hypochlorite group and (D) citric acid group.

The qualitative assessment of SEM images indicates the presence granular debris on the dentin wall and distinct interfacial gaps in the sealer dentin interface in the control group (Fig. 2). The remaining groups showed no uniform penetration of the sealer into the adhesive interface with smaller gaps (Fig. 3).

## Discussion

While Ca(OH)_2_ is invaluable during RCT, its thorough removal before permanent obturation is critical to ensure the adhesion and sealing ability of the root canal filling (De Almeida Barbosa et al., 2024). Utilizing effective irrigation protocols can mitigate these challenges and enhance clinical outcomes. While previous research has explored the effects of Ca(OH)_2_ on dentin and its impact on sealer adhesion (Vilela et al., 2011; Amin, Seyam & El-Samman, 2012; Barbizam et al., 2008), there is no consensus regarding the optimal irrigation protocol for its removal (Savaris et al., 2024). Moreover, limited studies have examined the relationship between Ca(OH)_2_ removal and the PBS between HCSSs and root dentin (Amin, Seyam & El-Samman, 2012). This study investigated the bond strength between HCSS and root dentin after using different irrigation protocols to remove Ca(OH)_2_. The results indicate that the null hypothesis was rejected, as the irrigation protocols for the Ca(OH)_2_ removal significantly influenced the PBS.

Glycolic acid, a relatively novel irrigation solution, demonstrated superior PBS, particularly in the coronal and apical thirds, compared to distilled water. GA has shown comparable or superior performance to EDTA in removing smear layers, with reduced cytotoxicity and biodegradability advantages (Dal Bello et al., 2019; Souza et al., 2024). Its ability to remove Ca(OH)_2_ residues effectively has been reported in previous studies (Keskin, Keleş & Sarıyılmaz, 2021; Yanık & Turker, 2024) and is attributed to its dual chelating and cleaning properties, which might enhance sealer adaptation. The selection of 5% glycolic acid in this study was based on findings from previous research reporting that this concentration achieves effective smear layer removal while minimizing dentin erosion (Dal Bello et al., 2019). Therefore, a 5% concentration was deemed to provide an optimal balance between cleaning efficacy and preservation of dentin integrity. Maintaining the structural integrity of dentin is critical for long-term endodontic success. Aggressive chelation, such as that caused by prolonged exposure to EDTA, can weaken dentin and increase susceptibility to fracture (Calt & Serper, 2002). Glycolic acid, with its lower erosive potential, preserves dentin’s biomechanical properties (Dal Bello et al., 2019; De Andrade Marafiga et al., 2022). Glycolic acid demonstrated similar PBS values, likely attributed to its milder demineralizing action and ability to preserve the dentin collagen matrix.

This finding supports earlier research highlighting the role of chelating agents, such as EDTA and citric acid, in improving dentin surface cleanliness and bond strength (Neelakantan et al., 2015). EDTA + NaOCl and citric acid also achieved significantly higher PBS compared to distilled water, consistent with studies reporting the effectiveness of these solutions in enhancing resin sealer penetration into dentinal tubules (Savaris et al., 2024). However, no significant difference was observed between these two groups, suggesting comparable efficacy in removing Ca(OH)_2_ residues. Chelators were reported to negatively affect HCSSs by disrupting the hydration process and chelating calcium ions necessary for hydroxyapatite formation (Lee et al., 2007), potentially compromising the material’s biocompatibility and hardness, while NaOCl has been reported to enhance the alkaline environment conducive to calcium silicate hydration (Asawaworarit et al., 2016).

The predominance of cohesive failures across all groups indicates that the sealer-dentin bond was stronger than the internal cohesive strength of the sealer. Cohesive failures occur when the fracture is within the material itself, rather than at the interface between the sealer and dentin (Nabavizadeh et al., 2022). In this study, cohesive failures were the predominant failure mode across all groups, indicating that the sealer-dentin bond was generally stronger than the internal cohesive strength of the sealer. This finding corroborates previous studies, which highlight its ability to form strong chemical bonds with dentin due to calcium ion release and hydroxyapatite precipitation along the adhesive interface (Abu Zeid & Alnoury, 2024; Reyes-Carmona, Felippe & Felippe, 2010). This finding is in accordance with the findings of previous studies (Huffman et al., 2009; Shokouhinejad et al., 2013) reporting the main mode of failure as cohesive failure after a 7-day storage period, which was applied in the present study. The lack of significant differences in failure modes across groups suggests that all tested irrigation protocols were capable of providing a clean dentin surface conducive to bonding. However, glycolic acid’s performance in preserving dentin integrity while achieving high bond strength positions it as a preferable option for clinical use.

HCSSs interact with dentinal walls to form an adhesive interface characterized by hydroxyapatite deposition and a mineralized interfacial layer (Atmeh et al., 2012). This occurs due to the release of calcium ions and the subsequent formation of calcium-phosphate structures when in contact with dentinal fluids. A mineralized layer with tag-like structures is frequently observed, which enhances the sealer’s sealing capacity and reduces microleakage. SEM analysis enabled the qualitative examination of the dentin-sealer interface. While SEM cannot chemically differentiate between dentin debris and residual Ca(OH)_2_, the presence of granular material and micro-gaps observed in the control group provided a morphological explanation for the significantly lower PBS values.

Glycolic acid’s effectiveness suggests it could serve as an alternative to traditional chelators such as EDTA and citric acid. Although this study was conducted under *in vitro* conditions, the findings provide some clinically relevant insights. The results suggest that irrigation protocols used for Ca(OH)_2_ removal may influence the bonding behavior of HCSSs to dentin. From a clinical perspective, this highlights the potential importance of selecting appropriate chelating agents during calcium hydroxide removal when bioceramic-based sealers are planned for obturation. The superior performance observed for glycolic acid suggests that it may represent a promising alternative to traditional EDTA. Given that HCSSs release calcium ions during hydration and establish chemical interaction with dentin *via* ion exchange and interfacial mineralization (Atmeh et al., 2012), the use of a milder chelator like glycolic acid, which effectively removes the smear layer and Ca(OH)_2_ without aggressively depleting the dentinal mineral content, may offer a strategic advantage in optimizing the adhesive interface of bioceramic sealers.

This study presents several limitations that must be considered when interpreting the findings. First, as an *in vitro* investigation, the experimental conditions cannot fully replicate the complex biological and functional environment of the root canal system *in vivo*. The evaluation of PBS and adhesive interface was performed after a 7-day incubation period. This does not account for the long-term stability of the sealer-dentin bond under clinical conditions, where aging and functional stresses could alter the bond integrity. The premixed HCSS used in this study was withdrawn from the market by its manufacturer after the experiments were completed. This does not undermine the generalizability of the mechanistic findings regarding the effect of irrigation protocols on HCSS and dentin interaction following Ca(OH)_2_, because chelation and hydration processes and dentin surface preparation are governed by brand-independent chemical principles. Nevertheless, studies employing other HCSS brands would strengthen the external validity of these findings. The reuse of the final shaping instrument during the Ca(OH)_2_ removal protocol was necessary to mechanically dislodge the medicament and confirm apical size, it may have superficially altered the canal geometry or created a secondary smear layer. Although this procedure was standardized across all groups, it could potentially confound the assessment of the pure chemical cleaning efficacy of the tested chelating agents. Since energy dispersive X-ray spectroscopy (EDS) was not performed, the residual particles observed at the interface could not be chemically identified as Ca(OH)_2_. Future studies should incorporate elemental analysis to correlate cleaning efficacy with bond strength directly.

### Conclusion

Irrigation protocol significantly influenced the PBS of a premixed HCSS to root dentin. All chelating regimens (17% EDTA + 5.25% NaOCl, 10% citric acid, and 5% glycolic acid) produced higher bond strength than distilled water. Glycolic acid yielded bond strength comparable to EDTA + NaOCl and citric acid, with the largest improvement observed in the coronal third. Failure modes were predominantly cohesive across groups.

## Supplemental Information

10.7717/peerj.21236/supp-1 Supplemental Information 1 Raw Data

## Additional Information and Declarations

### Data Availability

The following information was supplied regarding data availability: The raw data is available in the Supplemental File.

## References

[ref-1] Abu Zeid ST, Alnoury AS (2024). Impact of bioactivity on push-out bond strength of AH plus bioceramic *versus* BC bioceramic root canal sealers. Applied Sciences.

[ref-2] AH Plus Brochure (2026). Dentsply Sirona, Product Information.

[ref-3] Al-Hiyasat AS, Alfirjani SA (2019). The effect of obturation techniques on the push-out bond strength of a premixed bioceramic root canal sealer. Journal of Dentistry.

[ref-4] Amin SAW, Seyam RS, El-Samman MA (2012). The effect of prior calcium hydroxide intracanal placement on the bond strength of two calcium silicate—based and an epoxy resin—based endodontic sealer. Journal of Endodontics.

[ref-5] Aminoshariae A, Primus C, Kulild JC (2022). Tricalcium silicate cement sealers: do the potential benefits of bioactivity justify the drawbacks?. Journal of the American Dental Association.

[ref-6] Asawaworarit W, Yachor P, Kijsamanmith K, Vongsavan N (2016). Comparison of the apical sealing ability of calcium silicate-based sealer and resin-based sealer using the fluid-filtration technique. Medical Principles and Practice.

[ref-7] Atmeh A, Chong E, Richard G, Festy F, Watson T (2012). Dentin–cement interfacial interaction: calcium silicates and polyalkenoates. Journal of Dental Research.

[ref-8] Baldasso FER, Roleto L, Silva VDD, Morgental RD, Kopper PMP (2017). Effect of final irrigation protocols on microhardness reduction and erosion of root canal dentin. Brazilian Oral Research.

[ref-9] Barbizam JVB, Trope M, Teixeira ÉC, Tanomaru-Filho M, Teixeira FB (2008). Effect of calcium hydroxide intracanal dressing on the bond strength of a resin-based endodontic sealer. Brazilian Dental Journal.

[ref-10] Bedran NR, Nadelman P, Magno MB, Neves AA, Ferreira DM, Pintor AVB, Maia LC, Primo LG (2020). Does calcium hydroxide reduce endotoxins in infected root canals? A systematic review and meta-analysis. Journal of Endodontics.

[ref-11] Calt S, Serper A (2002). Time-dependent effects of EDTA on dentin structures. Journal of Endodontics.

[ref-12] Dal Bello Y, Porsch HF, Farina AP, Souza MA, Silva EJNL, Bedran-Russo AK, Cecchin D (2019). Glycolic acid as the final irrigant in endodontics: mechanical and cytotoxic effects. Materials Science and Engineering: C.

[ref-13] De Almeida Barbosa M, Meyfarth SRS, Tomazinho FSF, Antunes LAA, Antunes LS, Gabardo MCL (2024). Does calcium hydroxide interfere with root canal sealer penetration in dentinal tubules? A systematic review and meta-analysis. Journal of Evidence-Based Dental Practice.

[ref-14] De Andrade Marafiga F, Barbosa AFA, Silva EJNL, Souza MA, Farina AP, Cecchin D (2022). Effect of glycolic acid and EDTA on dentin mechanical properties. Australian Endodontic Journal.

[ref-15] Da Silva JM, Silveira A, Santos E, Prado L, Pessoa OF (2011). Efficacy of sodium hypochlorite, ethylenediaminetetraacetic acid, citric acid and phosphoric acid in calcium hydroxide removal from the root canal: a microscopic cleanliness evaluation. Oral Surgery, Oral Medicine, Oral Pathology, Oral Radiology, and Endodontology.

[ref-16] De Souza LC, Neves GST, Kirkpatrick T, Letra A, Silva R (2023). Physicochemical and biological properties of AH plus bioceramic. Journal of Endodontics.

[ref-17] Donnermeyer D, Bürklein S, Dammaschke T, Schäfer E (2019). Endodontic sealers based on calcium silicates: a systematic review. Odontology.

[ref-18] El-Ma’aita AM, Qualtrough AJ, Watts DC (2013). The effect of smear layer on the push-out bond strength of root canal calcium silicate cements. Dental Materials.

[ref-19] Gillen BM, Looney SW, Gu L-S, Loushine BA, Weller RN, Loushine RJ, Pashley DH, Tay FR (2011). Impact of the quality of coronal restoration versus the quality of root canal fillings on success of root canal treatment: a systematic review and meta-analysis. Journal of Endodontics.

[ref-20] Huffman B, Mai S, Pinna L, Weller R, Primus C, Gutmann J, Pashley DH, Tay FR (2009). Dislocation resistance of ProRoot endo sealer, a calcium silicate-based root canal sealer, from radicular dentine. International Endodontic Journal.

[ref-21] (2012).

[ref-22] Keskin C, Keleş A, Sarıyılmaz Ö (2021). Efficacy of glycolic acid for the removal of calcium hydroxide from simulated internal resorption cavities. Clinical Oral Investigations.

[ref-23] Lee Y-L, Lin F-H, Wang W-H, Ritchie H, Lan W-H, Lin C-P (2007). Effects of EDTA on the hydration mechanism of mineral trioxide aggregate. Journal of Dental Research.

[ref-24] Nabavizadeh M, Sobhnamayan F, Sedigh-Shams M, Liaghat S (2022). Comparison of the push-out bond strength of ah plus sealer to dentin after using different herbal irrigation solutions as the final rinse. PLOS ONE.

[ref-25] Neelakantan P, Sharma S, Shemesh H, Wesselink PR (2015). Influence of irrigation sequence on the adhesion of root canal sealers to dentin: a fourier transform infrared spectroscopy and push-out bond strength analysis. Journal of Endodontics.

[ref-26] Prado M, Simão RA, Gomes BP (2013). Effect of different irrigation protocols on resin sealer bond strength to dentin. Journal of Endodontics.

[ref-27] Reyes-Carmona JF, Felippe MS, Felippe WT (2010). The biomineralization ability of mineral trioxide aggregate and portland cement on dentin enhances the push-out strength. Journal of Endodontics.

[ref-28] Sanz JL, López-García S, Rodríguez-Lozano FJ, Melo M, Lozano A, Llena C, Forner L (2022). Cytocompatibility and bioactive potential of ah plus bioceramic sealer: an *in vitro* study. International Endodontic Journal.

[ref-29] Savaris JM, Isoton JC, Fluck BF, Tedesco M, Bortoluzzi EA, Garcia LdFR, Teixeira CdS (2024). Comparative analysis of AH plus bond strength to root canal dentin and adhesive interface quality after calcium hydroxide removal using different irrigation protocols. Journal of Endodontics.

[ref-30] Shokouhinejad N, Gorjestani H, Nasseh AA, Hoseini A, Mohammadi M, Shamshiri AR (2013). Push-out bond strength of Gutta-percha with a new bioceramic sealer in the presence or absence of smear layer. Australian Endodontic Journal.

[ref-31] Souza MA, Bischoff KF, Ricci R, Bischoff LF, Reuter E, Gomes NS, Hofstetter MG, Santos EW, Weissheimer T, So MVR, Da Rosa RA, De Figueiredo JAP, Palhano HS, Bello YD (2024). Glycolic acid and ultrasonic activation: effects on smear layer removal, dentin penetration, dentin structure and bond strength of the root dentin filling material. Journal of Clinical and Experimental Dentistry.

[ref-32] Vilela DD, Neto MM, Villela AM, Pithon MM (2011). Evaluation of interference of calcium hydroxide-based intracanal medication in filling root canal systems. Journal of Contemporary Dental Practice.

[ref-33] Xie CZ, Healy T, Russell J (2007). EDTA in the environment: with special reference to the dairy industry. International Journal of Environment and Waste Management.

[ref-34] Yanık D, Turker N (2024). Glycolic acid on push-out bond strength of fiber post and smear removal: an *in vitro* study. Odontology.

